# Experimental analysis of multiple factors on hydraulic fracturing in coalbed methane reservoirs

**DOI:** 10.1371/journal.pone.0195363

**Published:** 2018-04-05

**Authors:** Fan Zhang, Geng Ma, Xiao Liu, Yunqi Tao, Dan Feng, Rui Li

**Affiliations:** 1 School of Energy Science and Engineering, Henan Polytechnic University, Jiaozuo, Henan, China; 2 State Key Laboratory for Coal Mine Disaster Dynamics and Control, Chongqing University, Chongqing, China; 3 Applied Technical College, China University of Mining and Technology, Xuzhou, Jiangsu, China; Delft University of Technology, NETHERLANDS

## Abstract

Hydraulic fracturing can improve the permeability of coalbed methane (CBM) reservoirs effectively, which is of great significance to the commercial production of CBM. However, the efficiency of hydraulic fracturing is affected by multiple factors. The mechanism of fracture initiation, morphology and propagation in CBM reservoirs is not clear and need to be further explored. Hydraulic fracturing experiment is an accurate tool to explore these mechanisms. The quantity of experimental coal rock is large and processing method is complex, so specimen made of similar materials was applied to replace coal rock. The true triaxial hydraulic fracturing experimental apparatus, 3D scanning device for coal rock section were applied to carry out hydraulic fracturing experiment. The results show that the initiation pressure is inversely proportional to the horizontal stress difference (Δσ) and positively related to fracturing fluid injection rate. When vertical stress (σ_v_) is constant, the initiation pressure and fracture width decrease with the increasing of Δσ. Natural fractures can be connected by main fracture when propagates perpendicular to the direction of minimum horizontal stress (σ_h_), then secondary fractures and fracture network form in CBM reservoirs. When two stresses of crustal stress are close and far different from the third one, the fracture morphology and propagation become complex. Influenced by perforations and filtration of fracturing fluid in specimen, fracturing fluid flows to downward easily after comparing horizontal well fracturing with vertical well fracturing. Fracture width increases with the decreasing of elastic modulus, the intensity of fracture is positively related with the elastic modulus of coal rock. The research results can provide theoretical basis and technical support for the efficient development of CBM.

## Introduction

Hydraulic fracturing is an effective method for improving the permeability and output of coalbed methane (CBM), which is of great significance for commercial exploitation of CBM[1–4]. The fracture initiation, morphology and propagation are important for the fracture network geometry, which influence the efficiency of hydraulic fracturing greatly in coal seam[5]. Therefore, it is of great significance to study the formation mechanism and geometry of hydraulic fracture in CBM reservoirs. The key factors affecting initiation pressure and fracture propagation include crustal stress, flow rate, volume of fracturing fluid, mechanical properties of coal rock, fracturing techniques and others[6–9].

Hydraulic fracturing experiments in the past decades have been conducted to study the effect of multiple factors on the mechanism of hydraulic fractures globally[10–14]. However, the mechanism of fracture initiation, morphology and propagation in CBM reservoirs is not very clear. In addition, the roughness, structure of fractured specimen greatly influence the coal rock’s mechanics, flow characteristics of fracturing fluid and other properties. Meanwhile, the quantitative study of fractured surface and fracture width is also very rare. So far, the technologies applying to testing hydraulic fractures include acoustic emission, CT tomography scanning, microscopic observation, shear wave diffraction, coloring agent and so on[15–20]. However, there are some difficulties and defects in the application by these technical means.

In this article, the true triaxial hydraulic fracturing system is applied to investigate the initiation pressure, fracture morphology and propagation through a series of hydraulic fracturing experiments. The roughness and superficial area are obtained by 3D scanning device for coal rock section. The experimental objects include (1) information of water pressure in experiments; (2) fracture morphology and propagation by observing fractured specimens, and (3) 3D scanning diagrams and superficial area of fractured specimen by non-contact scanning. The research results can provide theoretical basis and technical support for the efficient development of CBM.

## Hydraulic fracturing experiment

### Experimental apparatus

**Fig 1:**
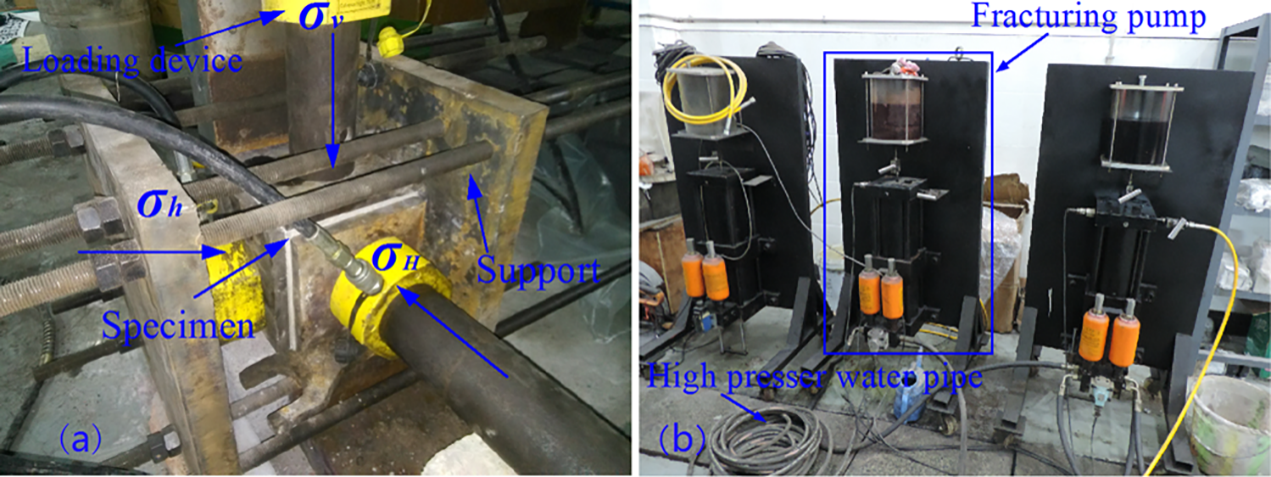
The physical experimental device. (a) true triaxial hydraulic fracturing system (b) fracturing fluid pump.

**Fig 2:**
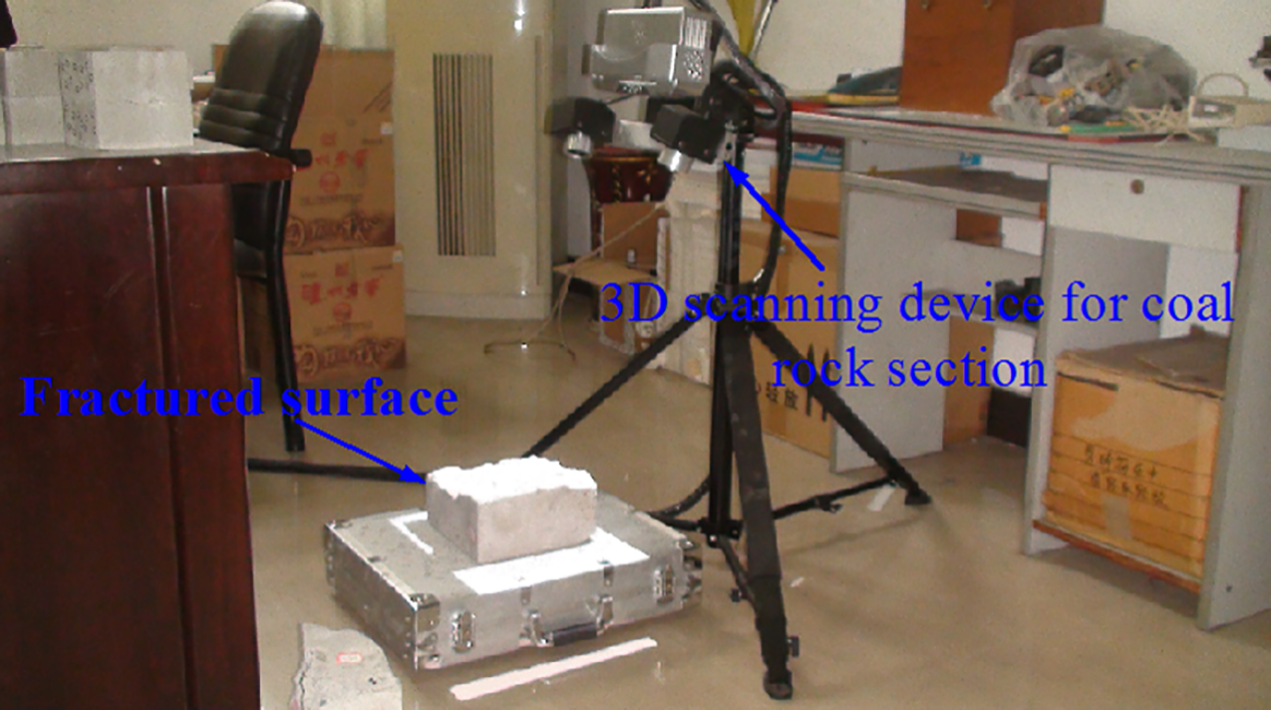
3D scanning device for coal rock section.

The physical experimental devices include true triaxial hydraulic fracturing system, fracturing fluid pump, 3D scanning device for coal rock section and other devices[21], as shown in Fig 1. The loading directions of crustal stresses are shown in Fig 1A and the stresses are loaded by manual hydraulic pump simultaneously. The 3D scanning device for coal rock section (Fig 2) can provide non-contact scanning for fractured specimen and large quantity data with high precision, thus the roughness and superficial area are obtained, which can make it more vivid to observe the fluctuation of fractured specimen, fracture morphology and propagation. The experiments of this study were carried out in public laboratory of Chongqing University and did not involve endangered or protected species.

### Specimen processing

#### Similar material selection

The coal rock in this article is collected from the No.4 coal seam of Xintian Coal Mine, Guizhou, China, where does not required specific permission. The quantity of experimental coal rock is large and processing method is complex, similar materials are used to mould specimen to simulate the coal rock. Based on similarity theory, the similar material for coal rock model is developed. Cement and gypsum are selected as cementing agent, and pulverized coal is applied to be aggregate, which can represent the destruction and mechanical properties of coal rock.

#### Mechanical properties of coal rock and similar materials

**Fig 3:**
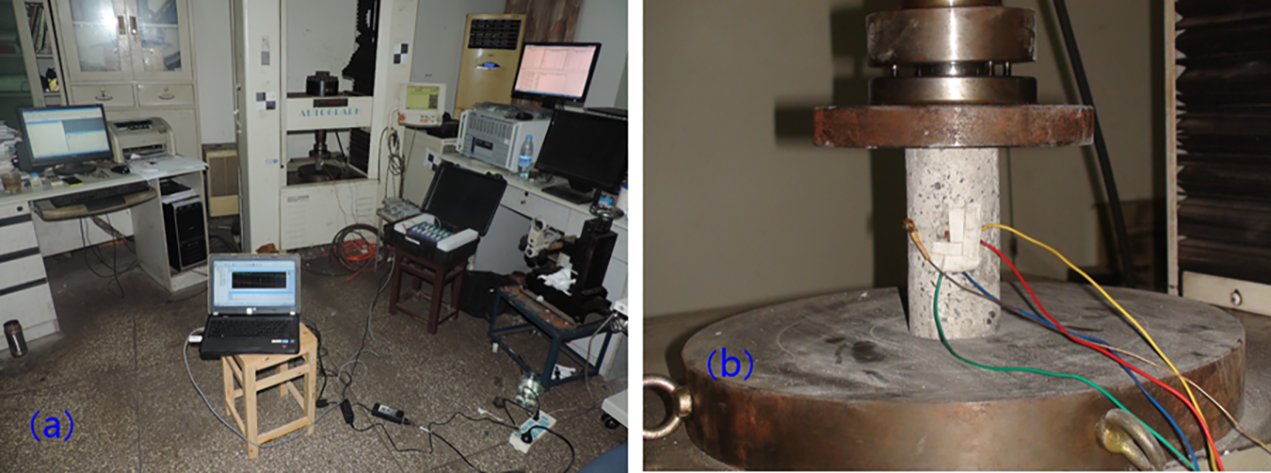
AGI 250 Electronic precision materials testing machine. (a) experimental devices (b) loading device.

To obtain the compressive strength (σ_c_), elastic modulus (E) and Poisson ratio (μ) of coal rock, we carried out uniaxial compression experiment. The device for uniaxial compression experiment was AG-I 250 kN Electronic Precision Materials Testing Machine, produced by SHIMADZU, as shown in Fig 3. The cylindrical specimens used in uniaxial compression tests had the diameter and height of 50 and 100 mm respectively. During the process of testing uniaxial compression strength, the axial deformation and radial deformation of the samples were tested by strain gage, the Poisson ratio was calculated in elastic stage by strain gage shaped T. The uniaxial compression experiment was controlled by displacement, the rate was 0.05 mm/min.

The firmness coefficient (f) was tested by means of counter balance, measuring cylinder, sieve, small hammer, funnel and other related devices. The steps for testing firmness coefficient of coal rock were as follows. Firstly, the coal rock was shaped into 20 mm and weight is 50 g. Secondly, firmness coefficient was tested by means of drop hammer for three times and coal rock was put into a diameter of 0.5 mm sieve for sieving. Finally, the coal powder was put into measuring cylinder, tapped gently to make it dense, the height of coal powder was read from measuring cylinder. The firmness coefficient of coal rock was obtained by

$$f=\frac{2n}{L}$$ (1)

where, f is the firmness coefficient, n is the number of shock, L is the height of powder read from measuring cylinder.

The mechanical parameters of coal rock were measured, its uniaxial compressive strength was 8.53 MPa, tensile strength was 0.63 MPa, elastic modulus was 0.82 GPa, Poisson ratio was 0.28, firmness coefficient was 0.80, respectively.

Whether similar simulation experiment can be conducted successfully depends on whether the mechanical properties of similar materials and coal rock are close. To make the mechanical parameters of coal rock close to the ones of similar materials, the mechanical parameters of similar materials were studied based on experimental program shown in Table 1 and tested by difference devices. The mechanical parameters of similar materials and coal rock are shown in Table 1.

**Table 1:** Mechanical parameters of similar materials and coal rock.

Group number	Ratio	σ_c_/MPa	E/GPa	μ	f
	cement:gypsum:pulverized coal				
A	1:1:1	3.01	0.55	0.22	0.61
B	1:1:1.5	5.41	0.75	0.17	0.64
C	1.5:1:1	5.47	0.65	0.35	0.65
D	1:2:1	3.13	0.66	0.28	0.58
E	2:1:1	5.36	0.95	0.25	0.82
Coal rock	—	8.53	0.82	0.28	0.80

Comparing the mechanical properties of similar materials with the ones of coal rock (as shown in Table 1), it can be seen that uniaxial compressive strength of similar materials of ratio B, C, E are close to 5.45 MPa. When the ratio of cement, gypsum, pulverized coal is 2, 1, 1, the elastic modulus, Poisson's ratio and firmness coefficient of similar materials are very close to the ones of coal rock, which shows that ratio E is accurate. From what has been discussed above, we can see that similar materials can replace coal rock to conduct hydraulic fracturing experiments when ratio E is chosen. Besides, the tensile strength of ratio E was tested and the tensile strength was 0.71 MPa, which was close to the one of coal rock (0.63 MPa).

#### Specimen processing

**Fig 4:**
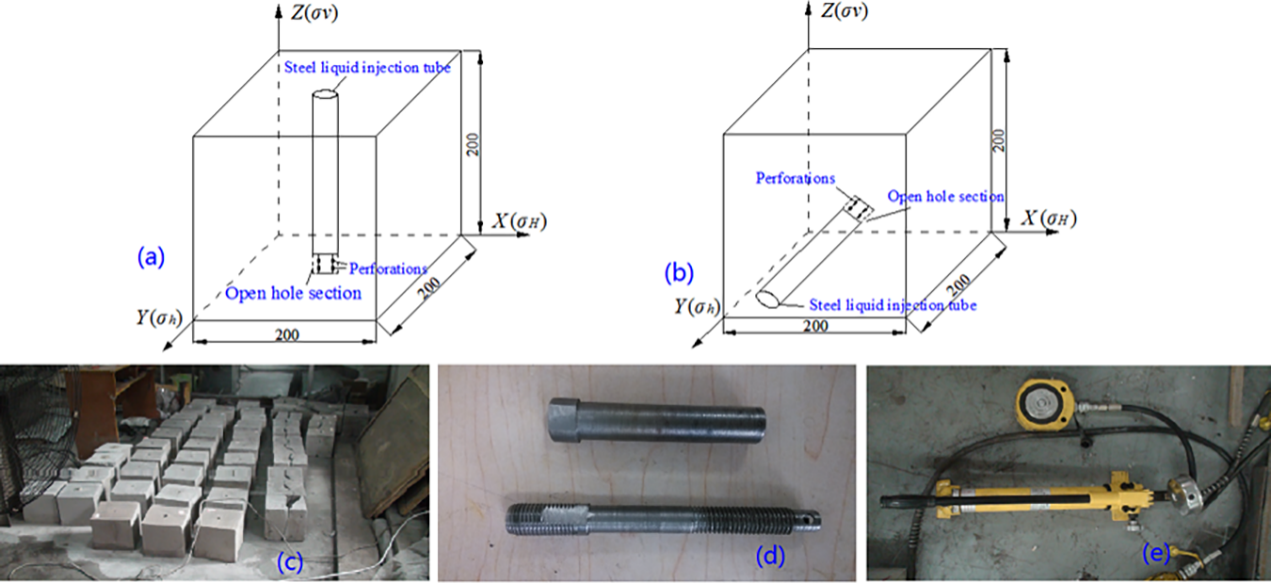
Stress loading device and specimen. (a) sketch map of vertical well fracturing (b) sketch map of horizontal well fracturing (c) specimen (d) steel liquid injection tube (e) manual hydraulic pump.

The specimens made of similar materials are processed into 200 mm cube in matrix. The vertical well fracturing pattern is shown in Fig 4A and horizontal well fracturing pattern is shown in Fig 4B. The directions of σ_H_ (maximum horizontal stress), σ_h_, σ_v_ and steel liquid injection tube are shown in Fig 4A and 4B. The steel liquid injection tube with a length of 115 mm is fixed into the eyehole by special chemical glue to simulate wellbore (Fig 4C). Besides, the steel liquid injection tube is processed with perforations symmetrically for pumping fracturing fluid, as shown in Fig 4D. Fig 4E is the stress loading device for σ_H_, σ_h_ and σ_v_.

### Experimental program

To explore the effect of crustal stress, natural fracture, fracturing direction, fracturing fluid injection rate and other factors on initiation pressure, fracture morphology and propagation in hydraulic fracturing experiments, the experimental program was designed as shown in Table 2.

**Table 2:** Experimental program for hydraulic fracturing experiment.

Specimen number	Crustal stress/MPa			Well direction	Natural fracture/ (vertical/horizontal)	Displacement/ (mL/s)
	σ_H_	σ_v_	σ_h_			
1#	1.00	0.50	0	vertical well	none	3.2
2#	1.25	0.50	0	vertical well	none	3.2
3#	1.50	0.50	0	vertical well	none	3.2
4#	2.00	0.50	0	vertical well	none	3.2
5#	2.50	0.50	0	vertical well	none	3.2
6#	2.50	1.00	0	vertical well	none	3.2
7#	2.50	1.50	0	vertical well	none	3.2
8#	2.50	2.00	0	vertical well	none	3.2
9#	1.50	0.50	0	vertical well	vertical fracture	3.2
10#	1.50	0.50	0	vertical well	horizontal fracture	3.2
11#	1.00	0.50	0	horizontal well	vertical fracture	3.2
12#	1.50	0.50	0	vertical well	none	4.0

In addition, to study the impact of mechanical properties on hydraulic fracturing, the specimen 13# made of similar materials based on the ratio of Group D (as shown in Table 1) and specimen 14# made of similar materials based on the ratio of Group E were applied to conduct hydraulic fracturing experiments with the flow rate of 3.2 mL/s and unstressed.

### Experimental procedure

To carry out hydraulic fracturing experiments, three dimensional stresses are applied to specimens based on the experimental program, as shown in Fig 4A and 4B. Then the fracturing fluid mixed red ink is pumped into steel liquid injection tube after the three dimensional stresses are loaded. The fracturing pump is not shut down until fracturing fluid flows out from specimen and the pressure data is recorded by the software entitling True Triaxial Testaid. The fracture morphology and propagation are observed by tracer and digital photos. In addition, the morphology, roughness, contour line and superficial area of fractured red zone are scanned by 3D scanning device for coal rock section.

## Experimental results and analysis

The initiation pressure and initiation time were obtained from the curve of water pressure. The statistical results of fractured red zone and 3D scanning results of fractured specimen were combined to analyze the initiation pressure, fracture morphology and propagation.

### Effects of crustal stress

#### Results and analysis of initiation pressure and initiation time

To study the effect of Δσ on initiation pressure and initiation time in CBM reservoirs, five groups of hydraulic fracturing experiments are carried out based on Table 2. No.1: Horizontal stress difference is 1.00 MPa (specimen 1#), No.2: Horizontal stress difference is 1.25 MPa (specimen 2#), No.3: Horizontal stress difference is 1.50 MPa (specimen 3#), No.4: Horizontal stress difference is 2.00 MPa (specimen 4#), No.5: Horizontal stress difference is 2.50 MPa (specimen 5#), the flow rate is 3.2 mL/s.

**Fig 5:**
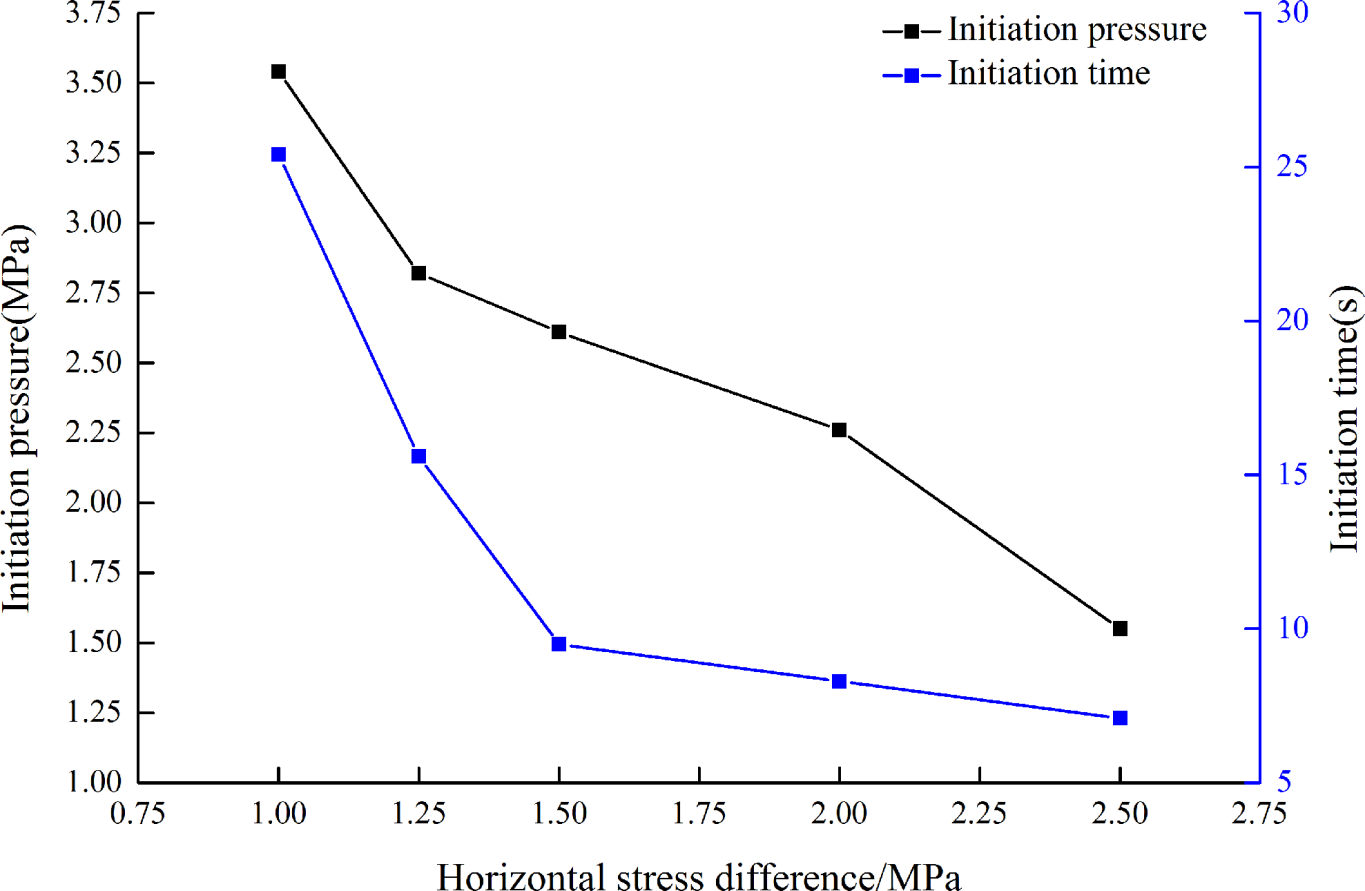
Initiation pressure and initiation time of different Δσ[21].

Three specimens were tested in hydraulic fracturing experiments under each condition. In Fig 5, five specimens were selected from fifteen ones to study the effect of horizontal stress difference on initiation time and initiation pressure. Fig 5 shows that with the increasing of Δσ, the initiation pressure and initiation time all decrease when σ_v_ is constant. That is to say, the varying tendency of initiation time and initiation pressure is inversely proportional to Δσ. Under each condition, the water pressure increases sharply once fracturing fluid is pumped into steel liquid injection tube. As water pressure reaches maximum water pressure, the initial fracture comes up. With the sustaining pumping of fracturing fluid, fractures continue propagating in specimen and the curve of water pressure fluctuates for the generation of new fractures.

To study the effect of σ_v_ on initiation pressure and initiation time, four groups of hydraulic fracturing experiments are conducted according to Table 2. No.1: Vertical stress is 0.50 MPa (specimen 5#), No.2: Vertical stress is 1.00 MPa (specimen 6#), No.3: Vertical stress is 1.50 MPa (specimen 7#), No.4: Vertical stress is 2.00 MPa (specimen 8#), the flow rate is 3.2 mL/s[22].

**Fig 6:**
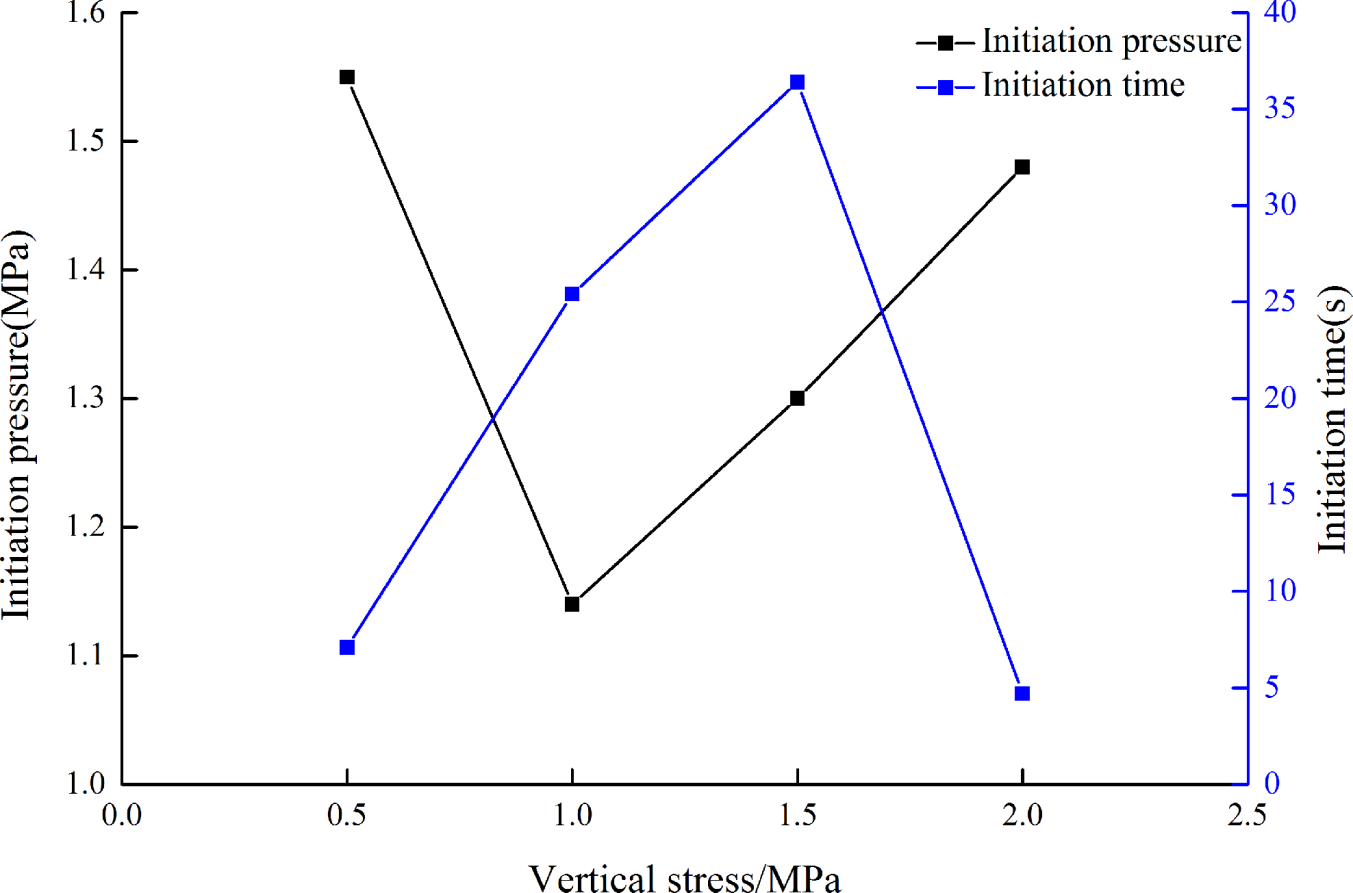
Initiation pressure and initiation time of different σ_v_[22].

Three specimens were tested in hydraulic fracturing experiments under each condition. In Fig 6, four specimens were selected from twelve ones to study the effect of vertical stress on initiation time and initiation pressure. Fig 6 presents that as Δσ is constant, the initiation pressure reduces firstly and then increases, the initiation time increases firstly and then decreases with the increasing of σ_v_. The tendency of initiation time and initiation pressure is different from the results of σ_v_.

#### Results and analysis of 3D scanning

3D scanning device for coal rock section is applied to extract the information of fractured specimen and 3D coordinates can be measured by non-contact scanning[21]. Five groups of hydraulic fracturing experiments are conducted based on Table 2, including No.1: Horizontal stress difference is 1.00 MPa (specimen 1#), No.2: Horizontal stress difference is 1.25 MPa (specimen 2#), No.3: Horizontal stress difference is 1.50 MPa (specimen 3#), No.4: Horizontal stress difference is 2.00 MPa (specimen 4#), No.5: Horizontal stress difference is 2.50 MPa (specimen 5#), the flow rate is 3.2 mL/s.

**Fig 7:**
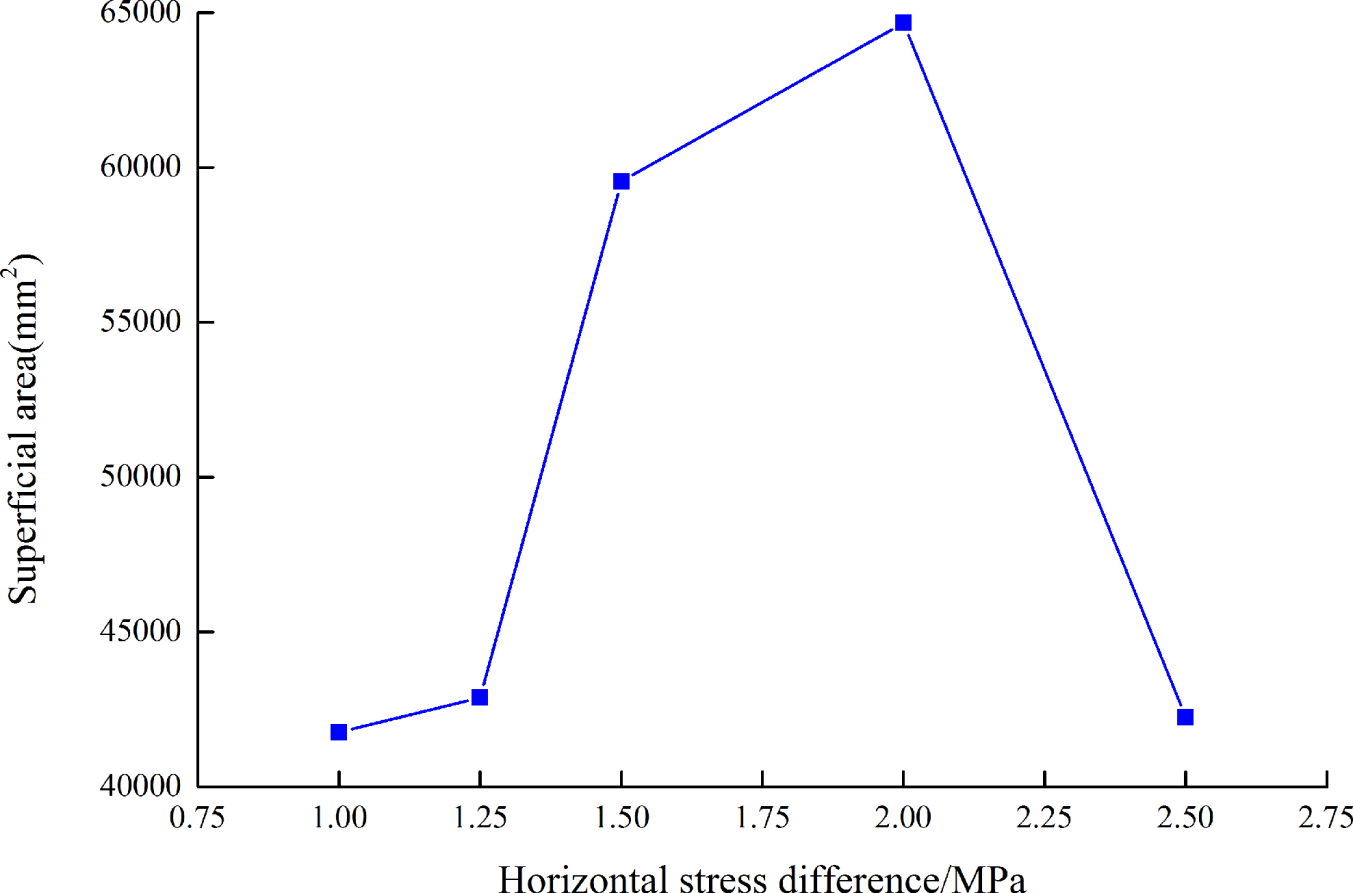
Curve of superficial area with Δσ[21].

The experimental results of 3D scanning present that the superficial area increases firstly and then reduces with the increasing of Δσ, as shown in Fig 7. When the Δσ increases from 1.00 MPa to 2.00 MPa, the superficial area of fractured specimen increases significantly, which is closely related to the tendency of initiation pressure and dynamic effect of fracturing fluid.

However, Fig 7 shows that the superficial area reduces as Δσ increases to 2.50 MPa. The reason is that the difference between σ_h_ and σ_v_ is 0.50 MPa, both of them are far smaller than σ_H_, the complexity of fracture morphology and propagation increases.

**Fig 8:**
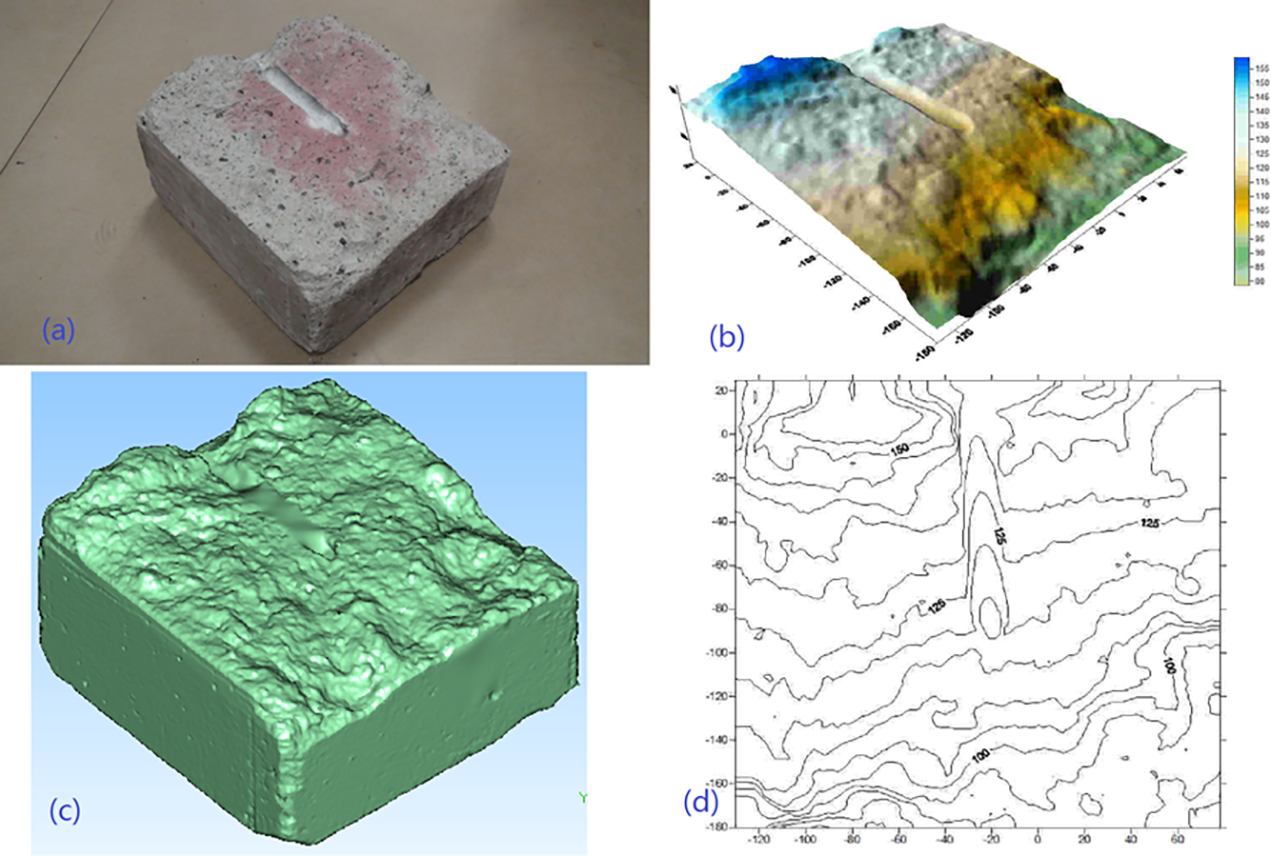
Schematic diagrams of fractured specimen. (a) fractured specimen (b) end view drawing (c) 3D scanning image (d) contour map.

In addition, the information extracted from fractured specimens can make it more vivid to observe the fracture morphology and propagation. For the Δσ of 2.00 MPa, the digital photo and results of 3D scanning are shown in Fig 8. Just as shown in Fig 8, the fracture morphology and propagation can be seen from fractured red zone (tracer),digital photos, end view drawing, 3D scanning image and contour map.

The experimental results present the 3D scanning image is more vivid and specific than just observing the tracer upon fractured specimen, as shown in Fig 8A and 8B. In Fig 8A, the red zone is fractured zone and the color varies from steel liquid injection tube to the edge of specimen, which can indicate the path of fracture propagation. Comparing Fig 8A, Fig 8B with Fig 8C, the fluctuation and fractured red zone of fractured surface can be obtained clearly to study the mechanism of fracture morphology and propagation. The experimental results show the edge of specimen is higher than the region of fracturing eyehole and the ordinate reduces below steel liquid injection tube, which is consistent with the information obtained in contour map. It can be seen that the end view drawing can provide reference and support for further analysis of fracture morphology and propagation upon fractured specimen. Contour map of fractured surface is obtained from three dimensional coordinates scanned by 3D scanning. Contour map can show the contour lines of fractured specimen, the range of contour lines is from 100 mm to 150 mm, as showed in Fig 8D. Furthermore, the fracturing sequence and fracture propagation can be inferred by comparing the fluctuation of fractured surface with tracer.

In summary, the function of 3D scanning technology in hydraulic fracturing experiment is shown as above, which can provide technical support and basis for the analysis of fracture width, fracture propagation, morphology and flow path of fracturing fluid.

### Effects of natural fractures

**Fig 9:**
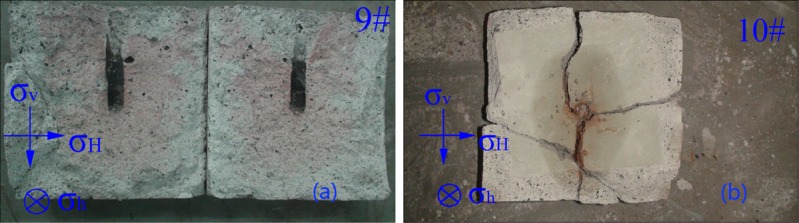
Morphology and fracture propagation of different natural fractures. (a) vertical artificial prefabricated fractures (b) horizontal artificial prefabricated fractures.

Two groups of experiments are designed and artificial prefabricated fractures (a kind of special paper with length, width and thickness of 130, 80 and 0.18 mm respectively) are inserted in specimen to simulate natural fractures, as shown in Fig 9. No.1: Horizontal stress difference is 1.50 MPa with vertical artificial prefabricated fractures (specimen 9#), No.2: Horizontal stress difference is 1.50 MPa with horizontal artificial prefabricated fractures (specimen 10#), the flow rate is 3.2 mL/s.

Fig 9 presents that the fractures propagate parallel to the direction of σ_H_ and path of fracture propagation is complex when fracturing fluid encounters with natural fractures. There are three kinds of propagation mechanisms as follows. To begin with, ringent natural fractures lead to the leak-off of fracturing fluid, fractures propagate along the surface of natural fracture to the end and then continue propagating parallel to the direction of σ_H_. Furthermore, fractures pass through natural fracture because of the powerful dynamic effect of fracturing fluid or fractures propagate along the surface of natural fracture, and then hydraulic fractures traverse natural fracture at weak point. Last but not least, hydraulic fractures stop propagating for large scale leak-off of fracturing fluid or ringent natural fracture.

It is found that the effect of different kinds of natural fractures on fracture morphology and propagation in hydraulic fracturing varies. The influence induced by natural fracture perpendicular to the direction of σ_h_ is more obvious. When hydraulic fractures meet with perpendicular natural fracture (specimen 9#), the dynamic effect of fracturing fluid and size of natural fracture are the key factors influencing the open of natural fractures. However, when hydraulic fractures meet with parallel natural fracture (specimen 10#), the large-scale parallel natural fracture restricts fracture propagation, which leads to low-intensity fracturing.

In conclusion, the fracture morphology and propagation are complex when fracturing fluid encounters natural fractures, main fracture is able to connect natural fractures in extending, thus fracture network forms in CBM reservoirs. The development degree and distribution of natural fractures affect the fracture morphology and propagation greatly.

### Effects of fracturing direction

Two groups of experiments are designed to explore the effect of fracturing direction on hydraulic fractures. No.1: Horizontal stress difference is 1.50 MPa with vertical well (specimen 9#), No.2: Horizontal stress difference is 1.00 MPa with horizontal well (specimen 11#), the flow rate is 3.2 mL/s.

**Fig 10:**
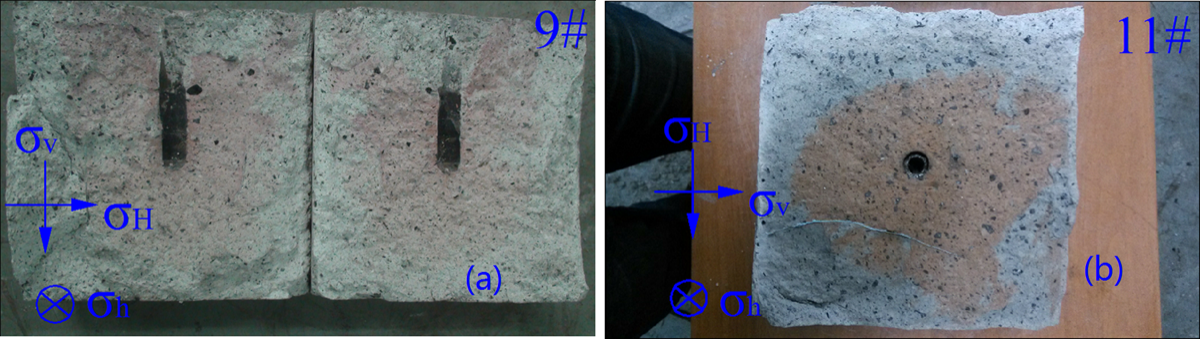
Fracture morphology and propagation of different fracturing direction. (a) vertical well (b) horizontal well.

In this article, the horizontal fracturing experiments is conducted by specimen 11#. For specimen 9# (vertical well fracturing) and specimen 11# (horizontal well fracturing), the perforations are parallel to the direction of σ_H_. The experimental results show that once fracturing fluid is pumped into steel liquid injection tube, the initiation fracture generates. The hydraulic fractures don’t stop propagating in specimens until fracturing fluid flows out from specimen. In specimen 9# (Fig 10A), we can see that the possibility of fractures propagating to two sides of perpendicular to the direction of σ_h_ is equal from the fractured red zone (tracer). While in specimen 11# (Fig 10B), it is found that the fracture propagates to the downward of specimen for the leak-off of fracturing fluid, resulting in declining dynamic effect of fracturing fluid and cease of fracture propagation.

### Effects of fracturing fluid injection rate

Two groups of experiments are designed as constant σ_v_. No.1: Horizontal stress difference is 1.50 MPa (specimen 3#), the flow rate is 3.2 mL/s; No.2: Horizontal stress difference is 1.50 MPa (specimen 12#), the flow rate is 4.0 mL/s.

**Fig 11:**
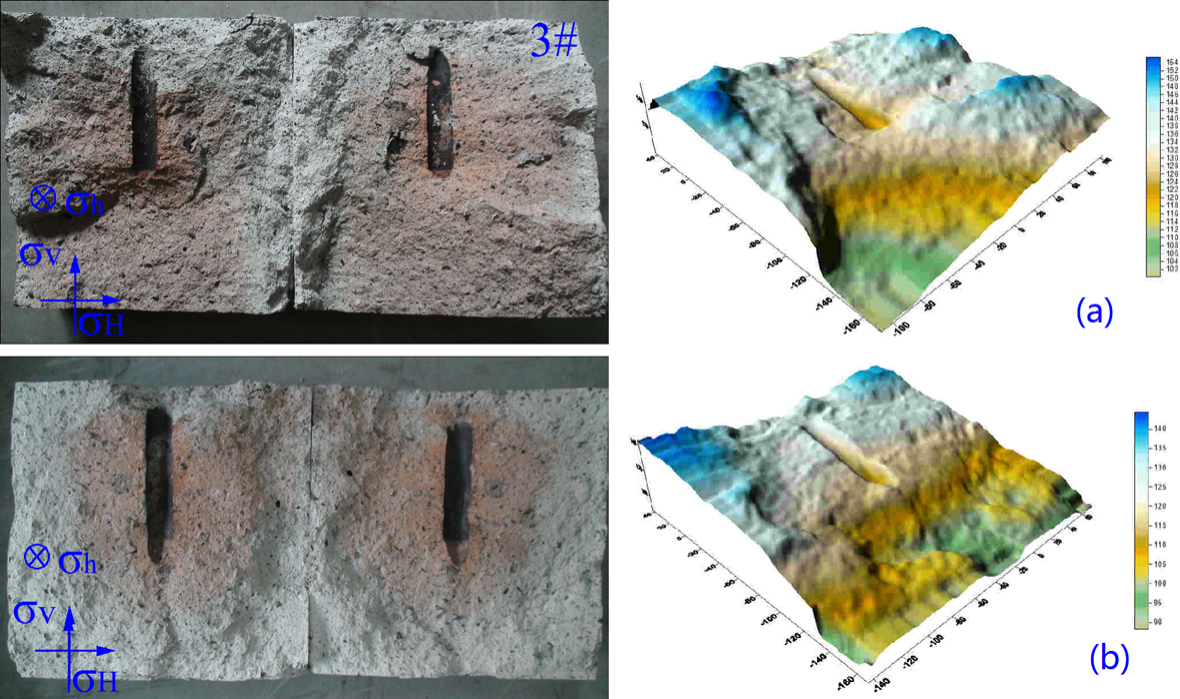
Fracture morphology and propagation of different fracturing fluid injection rate. (a) fracture morphology and propagation as flow rate is 3.2 mL/s (b) fracture morphology and propagation as flow rate is 4.0 mL/s.

Low fracturing fluid injection rate in hydraulic fracturing experiment leads to relatively low initiation pressure, as shown in Fig 11A. Fig 11A shows the fractured surface is rough and undulant. The reason is that the specimen is made of pulverized coal, which results in anisotropism in specimen. When fracturing fluid meets with large-grained pulverized coal, fractures propagate along the surface of large-grained coal. The CBM reservoirs have low permeability and various kinds of natural fractures, the fracturing fluid pumped into steel liquid injection tube can lead to the generation of initiation fracture and then connect natural fractures, resulting in the formation of secondary fracture. With continuous pumping of fracturing fluid, more natural fractures are connected in extension. For low fracturing fluid injection rate of vertical well, the fluid flows can make main fracture contact with more natural fractures and multiple fractures form, which is beneficial to the establishing of fracture network.

High fracturing fluid injection rate leads to extremely high initiation pressure and powerful dynamic effect of fracturing fluid, resulting in a straight fractured surface, as shown in Fig 11B. The fractures propagate perpendicular to the direction of σ_h_, which isn’t beneficial to the complexity of fractures and possibility of forming fracture network reduces. For high fracturing fluid injection rate, the natural fractures are not connected by main fracture and fracturing fluid flows out from the edge of specimen at shortest distance.

### Effects of mechanical properties

Two groups of experiments are designed to investigate the effect of mechanical properties on initiation pressure and fracture width. No.1: Stress is 0 MPa (specimen 13#), No.2: Stress is 0 MPa (specimen 14#), the flow rate is 3.2 mL/s.

**Fig 12:**
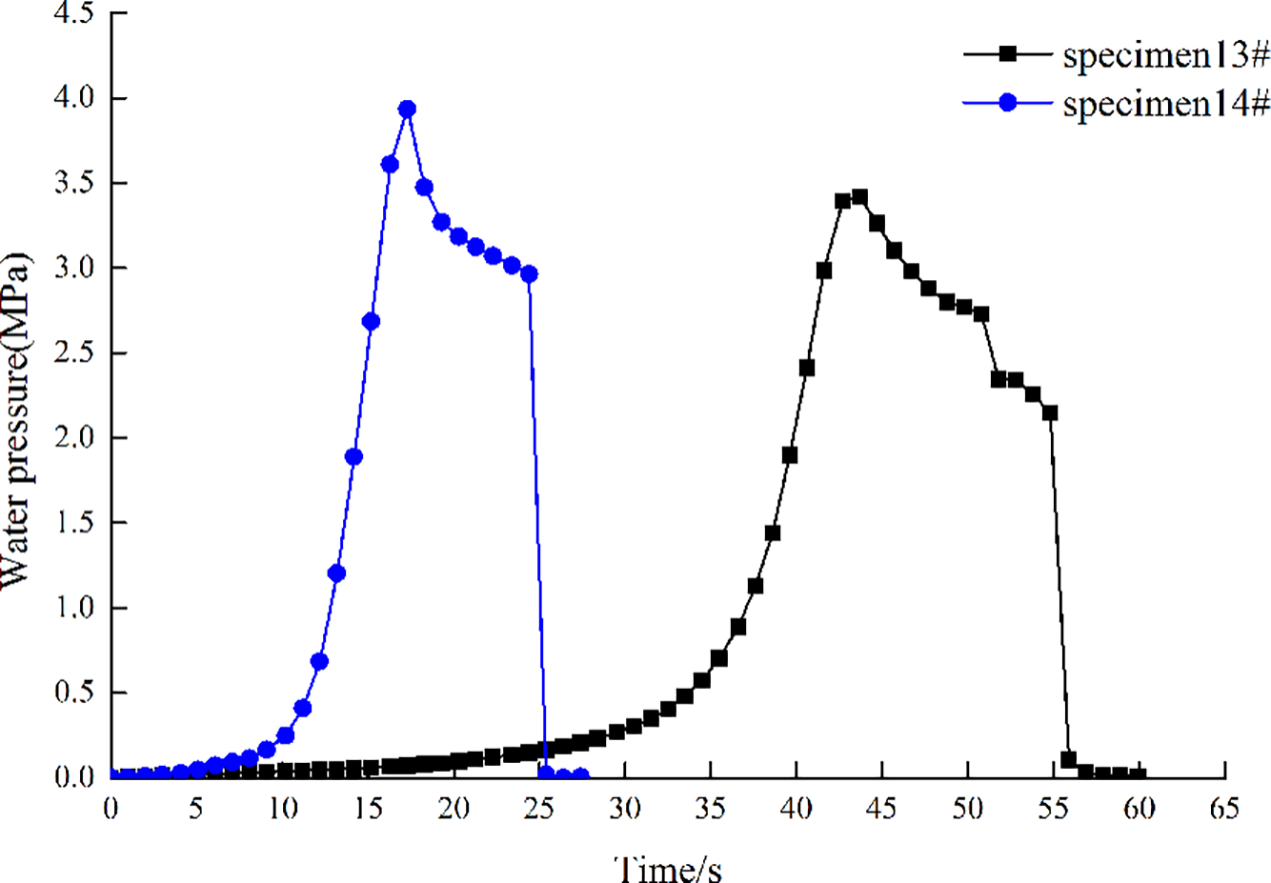
Curve of water pressure with time.

**Fig 13:**
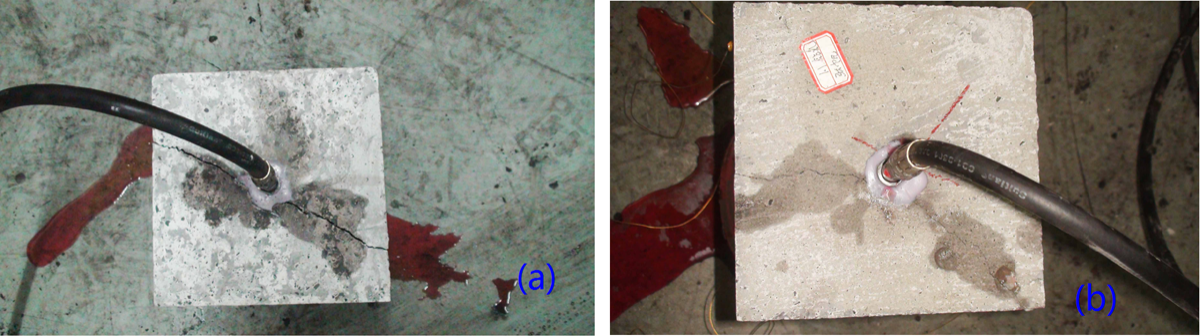
Fracture width and morphology of different specimens. **(a)** specimen 13# (b) specimen 14#.

Table 1 shows the elasticity modulus of specimen 13# is smaller than the one of specimen 14. Fig 12 presents that the initiation pressure of specimen 13# is smaller than the one of specimen 14, while the initiation time of specimen 13# is longer than the one of specimen 14. Fig 13 shows that the fracture width of specimen 13# is larger than the one of specimen 14. The experimental results present that the initiation pressure is positively related with the elastic modulus of coal rock, the initiation time and fracture width are inversely proportional to the elastic modulus of coal rock.

## Discussion

In CBM reservoirs, the natural fractures (butt cleat, face cleat and fractures) and permeability of coal rock are the key factors influencing the commercial development of CBM. The permeability of CBM reservoir is greatly affected by natural fractures. While hydraulic fracturing technology is adopted in CBM reservoirs, the geometry (including length, width and height) of fractures is of great significance to the optimization design of hydraulic fracturing.

When hydraulic fracturing technology is applied to improve the permeability of CBM reservoirs, hydraulic fractures are formed under the action of fracturing fluid. To date, the breakdown criteria is widely used to test the initiation pressure of coal rock. The initiation pressure can be obtained by

$$p=3\sigma_{h}‑\sigma_{H}+p_{f}+T_{0}$$ (2)

where, p is the initiation pressure, σ_h_ is the minimum horizontal stress, σ_H_ is the maximum horizontal stress, p_f_ is the tensile strength of coal rock, T_o_ is the pipeline resistance.

The above equation indicates that when σ_v_ and σ_h_ are constant, the initiation pressure decreases with the increasing of σ_H_, which is accordance with the experimental results. Thus the dynamic effect of fracturing fluid increases, fracture width reduces and fracture length increases with the increasing of Δσ.

**Fig 14:**
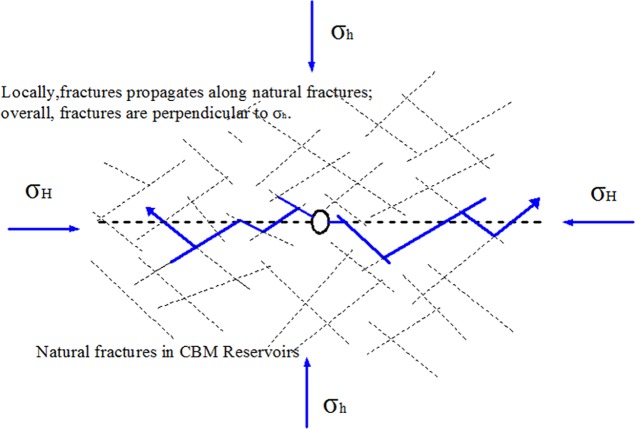
Fracture propagation in CBM reservoirs.

As fracturing fluid is pumped into CBM reservoirs, initiation fracture forms near eyehole and propagates perpendicular to the direction of σ_h_. With the continuous pumping of fracturing fluid, the initiation fracture can easily turn to the direction of natural fractures or the region where resistance is small. However, the whole tendency of fracture propagation doesn’t change for the generation of secondary fractures and the connection of former fracture with natural fractures, as shown in Fig 14. The fractures stop propagating when meet with large scale natural fractures, resulting in large scale leak-off of fracturing fluid and decreasing of fracture length. Subsequent fracturing fluid gathers together on the tip of fractures, which leads to the increasing of fracture width. As discussed above, high fracturing fluid injection rate has powerful dynamic effect on fracture propagation and straight fracture easily forms. While low fracturing fluid injection rate can make former fractures connect natural fractures, which is beneficial to the formation of fracture network. As shown in Fig 14, fractures propagates along natural fractures locally and the whole tendency of fracture propagation is perpendicular to the direction of σ_h_.

## Conclusions

The true triaxial hydraulic fracturing experiments of multiple factors are conducted to investigate the morphology and fracture propagation in specimen. The superficial area, roughness and vivid morphology upon fractured specimen are clearly observed by 3D scanning device for coal rock section. Some conclusions have been drawn:

1. The initiation pressure and initiation time are mainly influenced by in situ stress, fracturing fluid injection rate and elasticity modulus of coal rock.
2. Hydraulic fractures propagate along natural fractures locally and the whole tendency of fractures is parallel to the direction of maximum horizontal stress in CBM reservoirs.
3. When the difference of two parameters of crustal stress is small and both of them are far bigger or smaller than the third one, the fracturing fluid deflects easily and the complexity of fractures increases, which is beneficial to the formation of fracture network.
4. 3D scanning device for coal rock section is used to extract the details of fractured specimen. The superficial area of fractured specimen is obtained through 3D scanning, which is proved to be related to horizontal stress difference.

## Supporting information

S1 Dataset (ZIP)
